# Anti-artifacts techniques for neural recording front-ends in closed-loop brain-machine interface ICs

**DOI:** 10.3389/fnins.2024.1393206

**Published:** 2024-05-09

**Authors:** Weijian Chen, Xu Liu, Peiyuan Wan, Zhijie Chen, Yi Chen

**Affiliations:** ^1^College of Microelectronics, Beijing University of Technology, Beijing, China; ^2^Beijing Academy of Blockchain and Edge Computing, Beijing, China

**Keywords:** biomedical, motion artifact, stimulation artifact, neural recording, closed-loop brain-machine interface

## Abstract

In recent years, thanks to the development of integrated circuits, clinical medicine has witnessed significant advancements, enabling more efficient and intelligent treatment approaches. Particularly in the field of neuromedical, the utilization of brain-machine interfaces (BMI) has revolutionized the treatment of neurological diseases such as amyotrophic lateral sclerosis, cerebral palsy, stroke, or spinal cord injury. The BMI acquires neural signals via recording circuits and analyze them to regulate neural stimulator circuits for effective neurological treatment. However, traditional BMI designs, which are often isolated, have given way to closed-loop brain-machine interfaces (CL-BMI) as a contemporary development trend. CL-BMI offers increased integration and accelerated response speed, marking a significant leap forward in neuromedicine. Nonetheless, this advancement comes with its challenges, notably the stimulation artifacts (SA) problem inherent to the structural characteristics of CL-BMI, which poses significant challenges on the neural recording front-ends (NRFE) site. This paper aims to provide a comprehensive overview of technologies addressing artifacts in the NRFE site within CL-BMI. Topics covered will include: (1) understanding and assessing artifacts; (2) exploring the impact of artifacts on traditional neural recording front-ends; (3) reviewing recent technological advancements aimed at addressing artifact-related issues; (4) summarizing and classifying the aforementioned technologies, along with an analysis of future trends.

## 1 Introduction of the artifacts in CL-BMI

### 1.1 Mechanism of BMI

Brain-machine interface (BMI) technology can restore communication and control to people who are severely paralyzed (McFarland et al., 2017). A typical BMI system consists of a power management module, a neural recording unit, a signal processing module to convert the neural signal recorded into a control signal, and an external effector device (such as haptic or tactile stimulator, etc.) to achieve stimulation. By acquiring neural signals at the front end of the damaged nerve can be divided into implantable data collection (electrode) and non-implantable data collection (functional magnetic brain imaging or functional near-infrared spectroscopy, etc.). Then BMI analysis them, and control the stimulator to stimulate the back end of the damaged nerve. This system will recover the lost function caused by the damage in the central or peripheral nerve system (Chen et al., 2022).

### 1.2 Mechanism of artifacts formation

*In vivo* neural recordings often encounter various artifacts, undermining the capture of essential neural signals, particularly in less constrained recording environments (Islam et al., 2012, 2014). These artifacts can be broadly classified into two types: motion artifacts (MA) and SA. MA arise from factors such as respiration, electrode impedance changes, and body movements, posing significant challenges for wearable biomedical recording devices (van Helleputte et al., 2014). Unique to CL-BMI, SA are generated by the concurrent stimulation and recording during closed-loop control. The stimulation pulses are coupled through the tissue impedance, resulting in the formation of SA at the input of the recording site (Chandrakumar and Marković, 2017a,b,c). These SA can be further categorized into common-mode artifacts (CMA) and differential-mode artifacts (DMA), depending on their impact on the neural signal (Pérez-Prieto et al., 2019).

**Figure 1:**
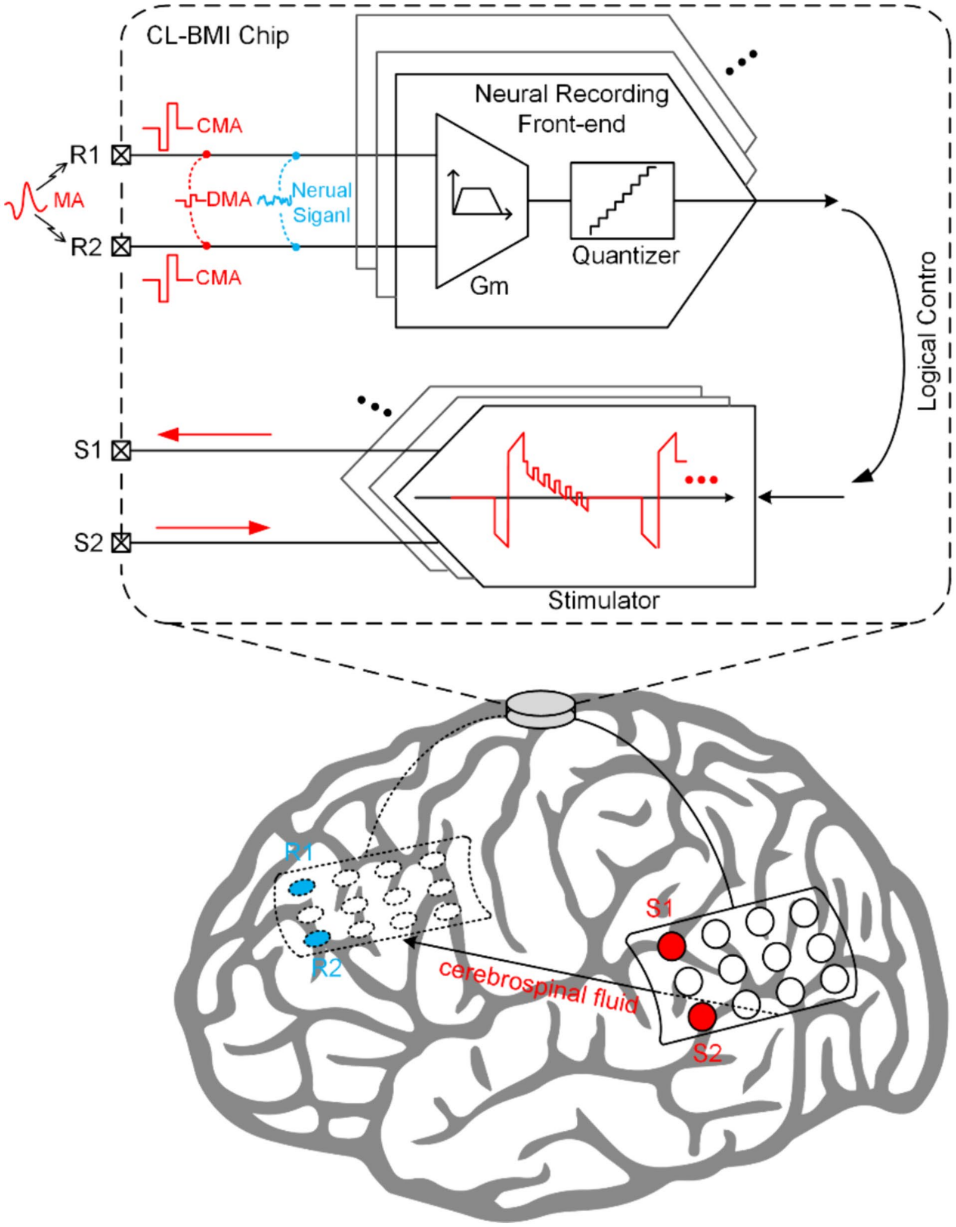
The formation mechanism of artifacts in CL-BMI interface.

As depicted in Figure 1, within the CL-BMI system, neural signals are accompanied by various artifact interference signals, which serve as input signals for NRFE. Consequently, during the design phase, designers must thoroughly evaluate the amplitude-frequency characteristics of diverse signals and the performance of neural recording front-ends to ensure a more informed and rational design approach.

### 1.3 Characteristics of artifact signals

Considering the formation mechanism of MA, it becomes evident that their amplitude and frequency are random, exhibiting a large dynamic range compared to the measured biopotential. Moreover, the bandwidth of MA may extend well within the biopotential signal bandwidth (Debbarma and Bhadra, 2022). Thus, the characteristics of MA necessitate evaluation through real-world experiments.

**Figure 2:**
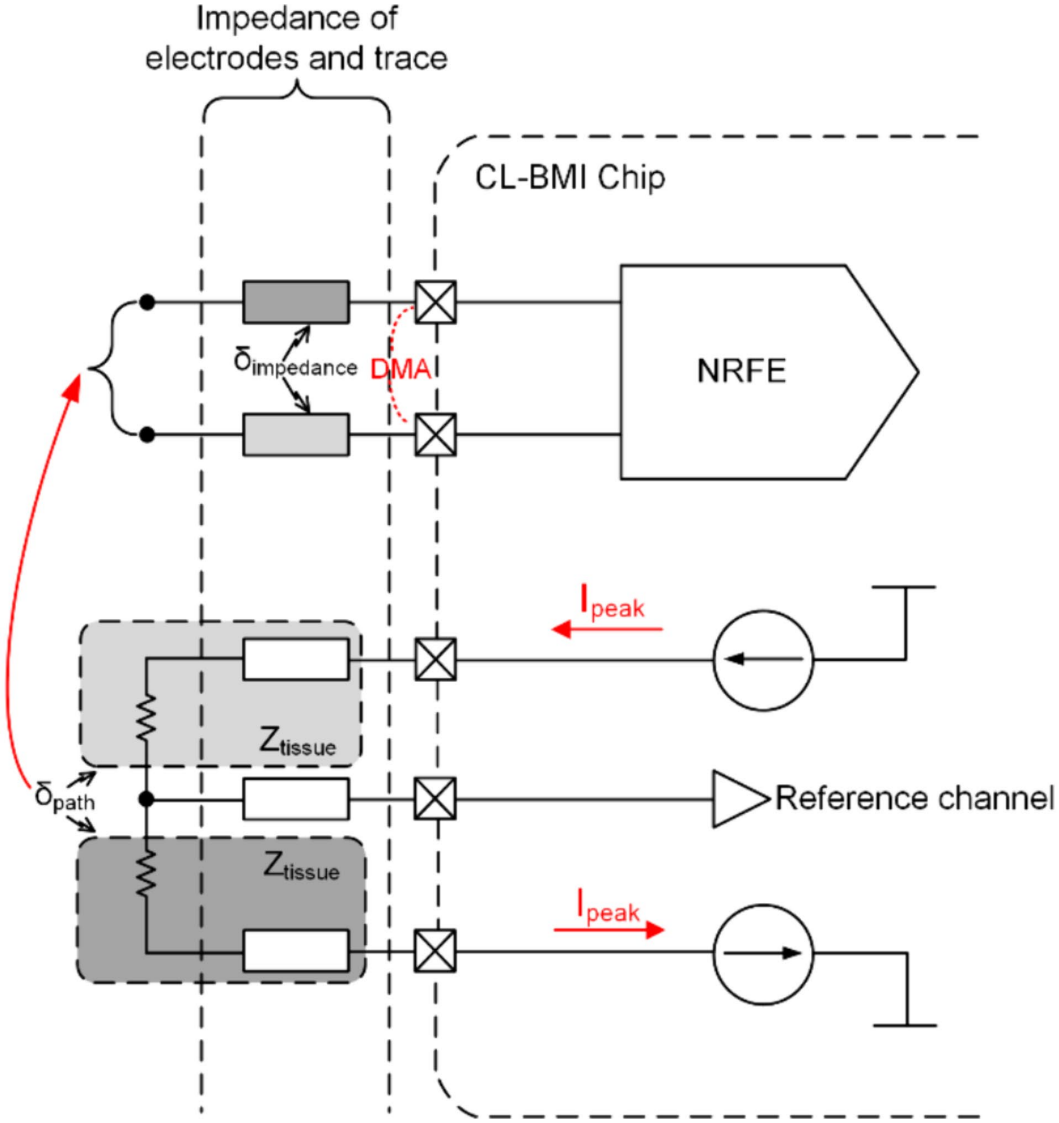
Simplified model of CL-BMI.

For the evaluation of SA, modeling of CL-BMI system can be employed, as depicted in Figure 2. This figure illustrates how the stimulation signal from a fully differential stimulator (FDS) generates SA through the direct conduction path of cerebrospinal fluid (CSF) and capacitive coupling from the electrode to the recording electronics, ultimately reaching the recording input site (Chandrakumar and Marković, 2017a,b,c). Notably, the FDS is utilized to mitigate artifact interference at the stimulation side (Liu et al., 2017; Pu et al., 2021), and SA occupy the same frequency band as the neural signal (Chandrakumar and Marković, 2017a,b,c). This model utilizes peak stimulation current amplitudes ($I_{\mathrm{peak}}$) and the impedance of the direct conduction path ($Z_{\mathrm{tissue}}$) to determine the peak-to-peak stimulation voltage swing ($V_{\mathrm{sigle}\;\mathrm{path}}$) for one path of the FDS.


$$V_{\mathrm{sigle}\;\mathrm{path}}=I_{\mathrm{peak}}\times Z_{\mathrm{tissue}}$$

Considering the path mismatch in the output of FDS due to process and complex environmental factors (with the mismatch coefficient denoted as $\delta_{\mathrm{path}}$), the peak-to-peak amplitude of FDS’s output (representing the CMA voltage, CMAV) is expressed by the formula:


$$\mathrm{CMAV}=V_{\mathrm{sigle}\;\mathrm{path}}\times \delta_{\mathrm{path}}$$

For recordings with fully differential inputs or those featuring reference channels, DMA arises due to impedance mismatch in the direct conduction path and electrodes (with the mismatch coefficient denoted as $\delta_{\mathrm{impedance}}$), and its voltage amplitude (DMAV) is denoted as:


$$\mathrm{DMAV}=\mathrm{CMAV}\times \delta_{\mathrm{impedance}}$$

Using recent publication (Li et al., 2023) on related application stimulators as an example, with $I_{\mathrm{peak}}≅3\;mA$ and $Z_{\mathrm{tissue}}=500\;\Omega$, assuming worst-case scenarios of $\delta_{\mathrm{path}}=$0.5 and $\delta_{\mathrm{impedance}}=0.1$, the resulting CMA and DMA are 750 mV and 75 mV, respectively.

The neural signals of interest are typically categorized into two types: local field potentials (LFP) and action potentials (AP). The frequency range of LFP is 1 to 100 Hz, with an amplitude of approximately 5 mV, while APs have a frequency range of 100 to 7 kHz, and an amplitude of approximately 100 μV (Harrison and Charles, 2003). As depicted in the figure, traditional NRFE may introduce interference or even annihilation of neural signals in practical applications due to the presence of artifact signals with amplitudes in the range of several hundred millivolts.

## 2 The influence of artifacts on prior research

**Figure 3:**
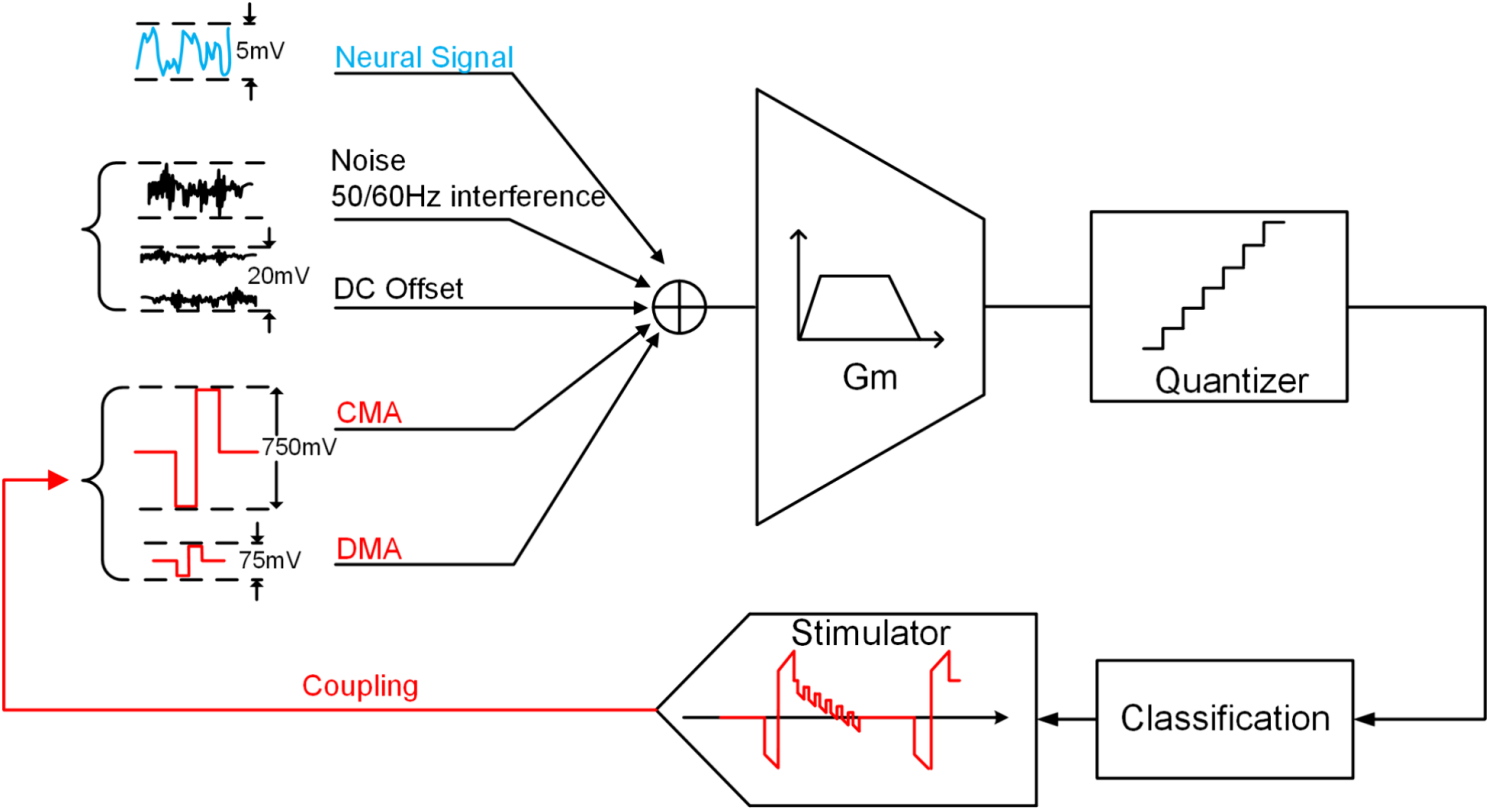
The concept of traditional neural recording with input interference.

Building on the analysis in the previous section, we can simplify the NRFE structure in CL-BMI, as depicted in Figure 3. Besides artifacts, the analog front end is susceptible to noise, offset voltages and 50/60 Hz interference, etc. (Verma et al., 2010). Although conventional neural recordings can eliminate these interferences, they may still be susceptible to artifacts that saturate the output.

### 2.1 Overview of the system requirement and the state-of-the-art

In the context of practical applications, designing NRFE system requires meeting various system requirements. Table 1, summarized from recent literature (Chandrakumar and Marković, 2017a,b,c; Liu et al., 2020), outlines these essential requirements.

**Table 1:** System requirement of neural recording.

Parameter	Required
Power (μW)	<5
BW (Hz)	1-5 k
In Band Noise ($\mu V_{rms}$)	4–8
DR (dB)	75
THD (dB)	−75
Input Range ($mV_{\mathrm{pp}}$)	100
CM Tolerance	Yes
Impedance @DC (Ω)	>1G
Area/Ch ($mm^{2}$)	<0.1

**Figure 4:**
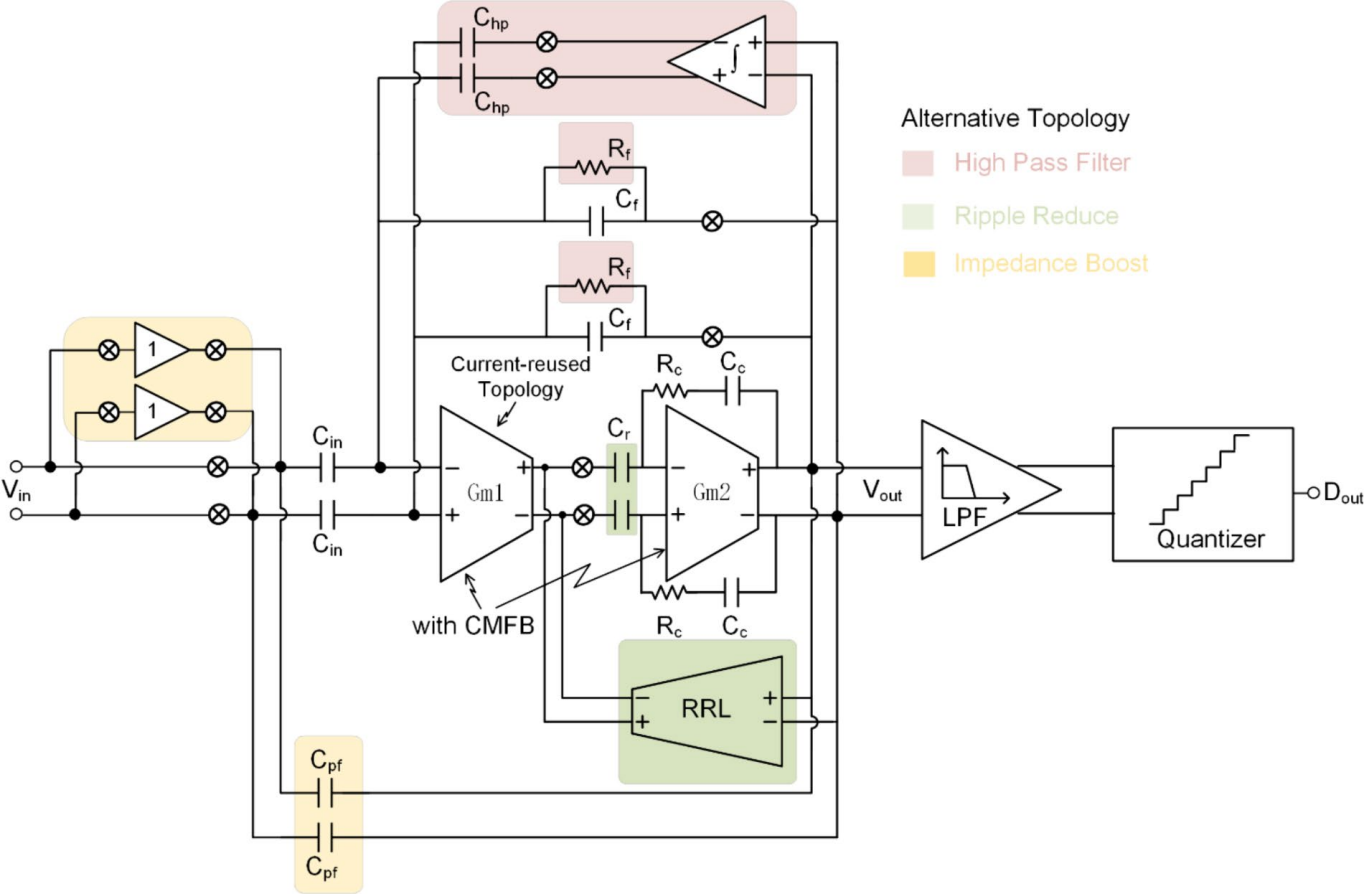
The state of the art of neural recoding.

Over the past two decades, extensive research on neural recording front-ends has been conducted, with continuous refinement (Oh et al., 2008; Zou et al., 2009; Fan et al., 2011; Zou et al., 2013; Mondal and Hall, 2017). Novel techniques and topologies of NRFE (illustrated in Figure 4) have emerged to optimize specific system requirements outlined in Table 1.

Figure 4 illustrates a complete neural recording front-end system featuring a capacitively-coupled chopper instrumentation amplifier (CCIA). The amplification and acquisition module of CCIA typically employs a two-stage operational amplifier structure, with the first stage commonly utilizing current-reuse technology to minimize the system’s input referred noise (IRN). To mitigate the DC offset introduced by the electrode, it is necessary to establish a high-pass cut-off frequency for the neural recording. To address ripple caused by the offset of the first stage of the neural recording, ripple reduction technology is necessary, achievable by connecting capacitors in series between two stages or introducing ripple reduction loop (RRL). Chopping technology significantly reduces the equivalent input impedance of the neural recording front-end and induces signal attenuation. Hence, impedance enhancement technology becomes necessary. Auxiliary charging path and positive feedback capacitors are both viable options.

While these techniques and structures are susceptible to artifacts, as highlighted in the previous analysis, they lay the foundation for the application of neural recordings in CL-BMI. By addressing specific system requirements, these advancements contribute to the ongoing progress in neural recording technology.

### 2.2 Tolerance of artifacts interference

As previously described, artifacts in neural recordings can be categorized into two types: CMA and DMA. This section will elaborate on their distinct impacts on traditional NRFE.

**Figure 5:**
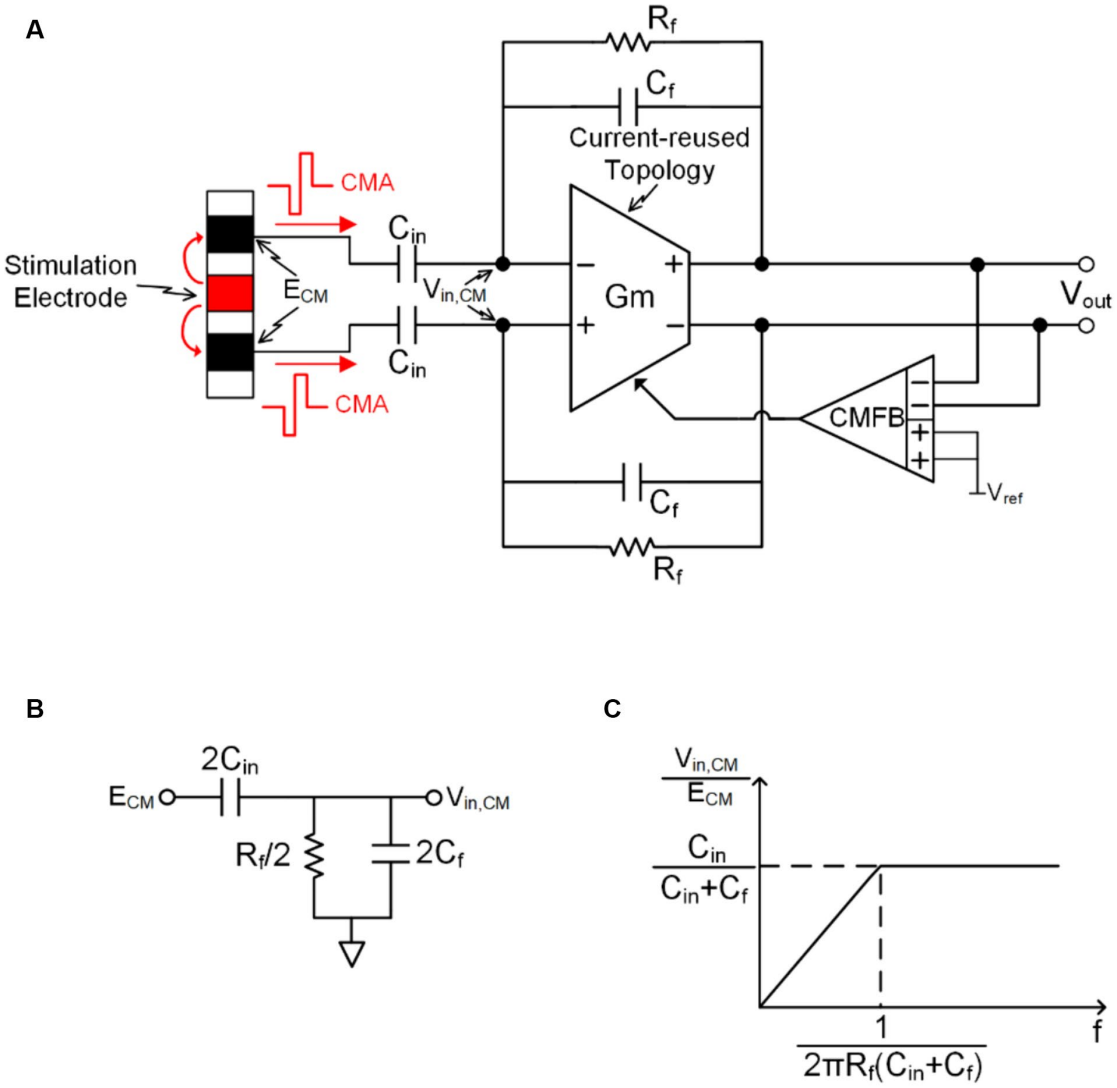
**(A)** CM response of conventional capacitive feedback neural recording. **(B)** The simplified CM response circuit. **(C)** Transfer function of the CM response.

Taking the fully differential capacitive negative feedback model illustrated in Figure 5A as an example, when the CMA, approximately 750 mV as discussed before, is present at the input of the recording electrode, it directly influences the signal at the output due to its nature as a common-mode (CM) swing signal. Fully differential amplifiers (FDA) typically utilize a common-mode negative feedback (CMFB) circuit to stabilize the output CM voltage (Zhang et al., 2018). Consequently, for CM signals, the output of FDA can be considered equivalent to AC ground. The equivalent CM circuit is depicted in Figure 5B. From this, we can derive the transfer function from the CM input signal at the electrode input end to the FDA input end. Therefore, the transfer function from the CM input signal at the electrode input site to the FDA input site can be derived.


$$\frac{V_{\mathrm{in},CM}}{E_{CM}}=\frac{\omega C_{1}R_{f}}{1+\omega \left(C_{1}+C_{f}\right)R_{f}}$$

where $V_{\mathrm{in},CM}$ is the CM input signal at the FDA input side and $E_{CM}$ is the CM input signal at the electrode input side.

The graph of the transfer function (Figure 5C) indicates that the model of CM voltage transfer across the recording electrodes can be considered as a first-order high-pass filter, with its corner frequency determined by $1/2\pi R_{f}\left(C_{\mathrm{in}}+C_{f}\right)$. In practical applications, this corner frequency is approximately 10 Hz. Therefore, CMA at the same frequency band as the neural signal can be transmitted to the input of FDA without attenuation. However, the amplitude of CMA far exceeds the input common-mode range (ICMR) of the FDA, resulting in saturation distortion in the FDA output. Despite employing amplifier topologies with a large common-mode input range, such as rail-to-rail or folded cascode topologies, total harmonic distortion (THD) at the neural recording front-end can still increase when artifacts are present at the input site. In cases of significant THD, the effective number of bits (ENOB) and signal-to-noise and distortion ratio (SNDR) of the quantization module will significantly deteriorate, leading to data conversion errors (Xu et al., 2018).

In general application neural recording front-ends, gains are typically set to more than 40 dB to amplify neural signals for quantification and processing. However, in the context of CL-BMI systems, DMA are also amplified by the neural recording front-end. As previously mentioned, the amplitude of DMA is approximately 75 mV, and maintaining the gain would also lead to output saturation.

It is evident that the introduction of CMA or DMA to the input site of NRFE leads to output saturation and distortion. However, considering their formation mechanism and signal nature, methods exist to shield the neural recording front-end from their interference.

## 3 Anti-artifacts technology

This section will introduce anti-artifact technologies developed over the past two decades. It is important to note that these technologies address a range of artifact issues, including MA and SA, each with distinct application backgrounds. However, despite the differences in application context, the consistent nature of the artifact signals allows for a unified approach in this section.

As mentioned previously, artifacts introduce nonlinear factors into neural recording front-ends due to their relatively high amplitude compared to neural signals and the limited dynamic range of the recording systems. Therefore, most anti-artifact technologies primarily focus on improving dynamic range and linearity and eliminating artifacts through back-end signal processing algorithms. Additionally, the periodic nature of artifacts is crucial for their effective elimination, further emphasizing the importance of advanced signal processing techniques in artifact mitigation strategies.

### 3.1 Current compensation technology utilized in rail-to-rail amplifiers

The most direct way to overcome common-mode artifact interference is to increase the input common-mode range (ICMR) to ensure that the neural amplifier can operate normally at higher common-mode input voltages. The rail-to-rail topology enables the widest possible input common-mode range, and a constant equivalent input transconductance $g_{m}$ can be achieved through current compensation techniques (Huang et al., 2022).

As mentioned in (Chandrakumar and Marković, 2017a,b,c), even if linearity can be preserved when the ICMR is larger than the amplitude of CMA, THD degraded when CMA was enabled. This distortion can be especially noticeable in systems with low noise and low power requirements such as neural recording. Therefore, simply improving ICMR is not advisable.

### 3.2 Moderate gain recording amplifier with high resolution ADC

One approach to increase dynamic range is to reduce the gain to prevent saturation of the neural recorder. However, if the gain is very low, it will bring challenges to the design of the ADC. This occurs due to the extremely low system gain inherent in this topology, resulting in an SNDR lower than the ADC in the same topology with a high-gain IA when the same amplitude signal is input. Considering that artifacts are amplified alongside neural signals, the ADC necessitates sufficiently high ENOB and SNDR to accommodate a broad signal input range.

Chandrakumar and Marković (2018) demonstrates a CCIA using a chopper-stabilized structure with a moderate gain of only 17.9 dB (≅8×). This makes the required ENOB of the proposed neural recording front-end larger than 15 bits and the required SNDR of 13 bits. Its ADC structure uses a continuous-time (CT) delta-sigma (DS) ADC structure, which has good power efficiency when ENOB>15 bits. The details of CT-DS technique will be described at the end in this section.

The disadvantage of this technology is that it is only immune to the influence of DMA, and its ability to tolerate DMA amplitude is limited due to the constraints of gain and dynamic range of the instrumentation amplifier (IA). In addition, the power consumption of this structure is higher than that of traditional high-gain IA and low-resolution ADC structures. This is because a low-gain IA cannot effectively mitigate the impact of quantization noise. Hence, opting for a high-resolution ADC is important, although at the expense of significantly elevated power consumption. (Jung et al., 2022a,b).

### 3.3 Adaptive gain control (AGC)

Since the artifact is generated by the stimulation current which is generated periodically in the CL-BMI system, the artifacts are also periodic signal. The stimulation cycle is very short compared to the entire neural signal acquisition period. Therefore, the performance of a fixed low-gain IA is not ideal during non-stimulation periods. The use of AGC technology allows the IA to perform varying gain adjustments according to the input signal amplitude to solve this issue (Delgado-Restituto et al., 2017).

**Figure 6:**
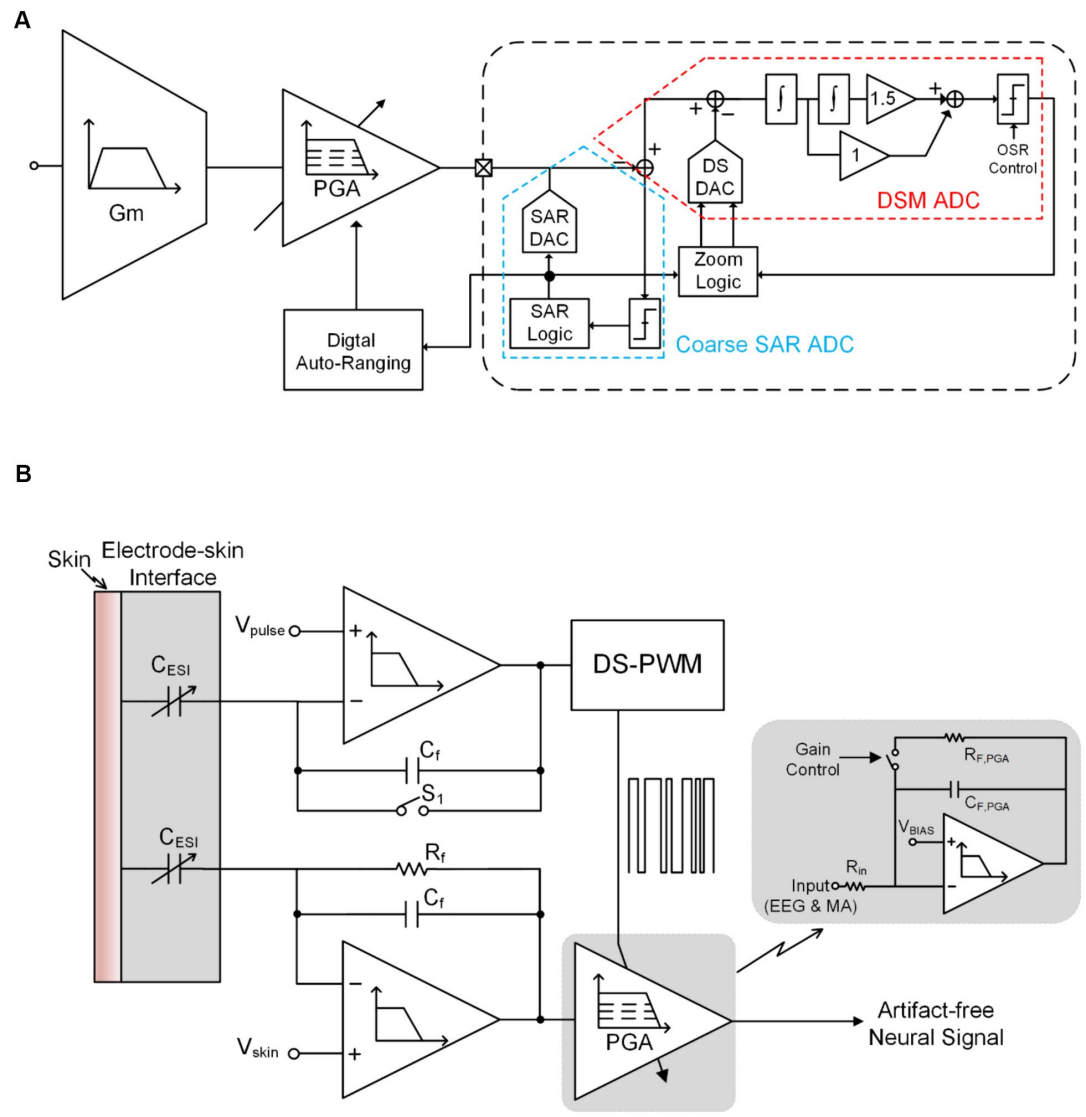
**(A)** AGC technique that utilizes SAR ADC reused in CT-DS modulator and DAR technology to control PGA gain. **(B)** the gain of low path’s (LP’s) PGA controlled by the up path’s (UP’s) output through a DS-pulse-width-modulation block.

Building upon the improvement of the previous technology, Figure 6A depicts AGC technique that utilizes SAR ADC reused in CT-DS modulator (CT-DSM) and digital auto-ranging (DAR) technology to control programmable gain amplifier (PGA) gain, thereby avoiding neural recording front-end saturation (Jung et al., 2021, 2022a,b). This system consists of a low gain instrumentation amplifier (IA), a PGA, a CT-DSM and a DAR block. The IA gain is fixed of 8 and the PGA gain is automatically controlled from 1 to 32 by DAR and CT-DSM depending on the input signal amplitude.

Figure 6B shows another AGC topology (but this topology is only applicable to MA), which the top-level block diagram of the gain of low path’s (LP’s) PGA controlled by the up path’s (UP’s) output through a DS-pulse-width-modulation (DS-PWM) block (Dabbaghian and Kassiri, 2023). The DS-PWM is operating with a 100 kHz frequency to guarantee well compensate the input artifact signal in real-time. The duty cycle of DS-PWM output (D) can be expressed as:


$$D=\alpha \;.\;\frac{C_{ESI}}{C_{f}}$$

Where α is constant determined by the relationship between $V_{P1}$, $V_{P2}$ (when PWM output (D) is zero, the DAC’s output is *VP*2 and when PWM output is VDD, the DAC’s output is *VP*1.), and $V_{REF}$; $C_{ESI}$ variations caused by random motion.

The gain of the overall neural recording front-end is independent of motion variation term $C_{ESI}$, and can be derived as:


$$A_{\mathrm{overall}}=A_{1}.A_{2}=\frac{C_{ESI}}{C_{f}}.\frac{R_{F,PGA}.C_{f}}{D.R_{\mathrm{in}}}=\frac{R_{F,PGA}}{R_{\mathrm{in}}}$$

where $R_{F,PGA}$ is the feedback resistor of PGA and $R_{\mathrm{in}}$ is the input resistor of PGA as shown in Figure 6B.

According to the description in (Pérez-Prieto, N. et al., 2019), there are two problems: Firstly, the larger the variable range of the PGA gain, the more complexity will increase in the digital reconstruction algorithm; secondly, according to the way the PGA changes the gain, it can be seen that the input-referred noise and bandwidth of the PGA will change under different gains.

### 3.4 Blanking

Due to the periodicity of SA, shutting off neural signal input during the neural stimulation cycle is an effective anti-artifact strategy. The technology of acquiring and processing neural signals outside of the stimulation cycle is collectively called blanking (Bi et al., 2020; Debarros et al., 2020; Huang et al., 2023; Qiu et al., 2023).

**Figure 7:**
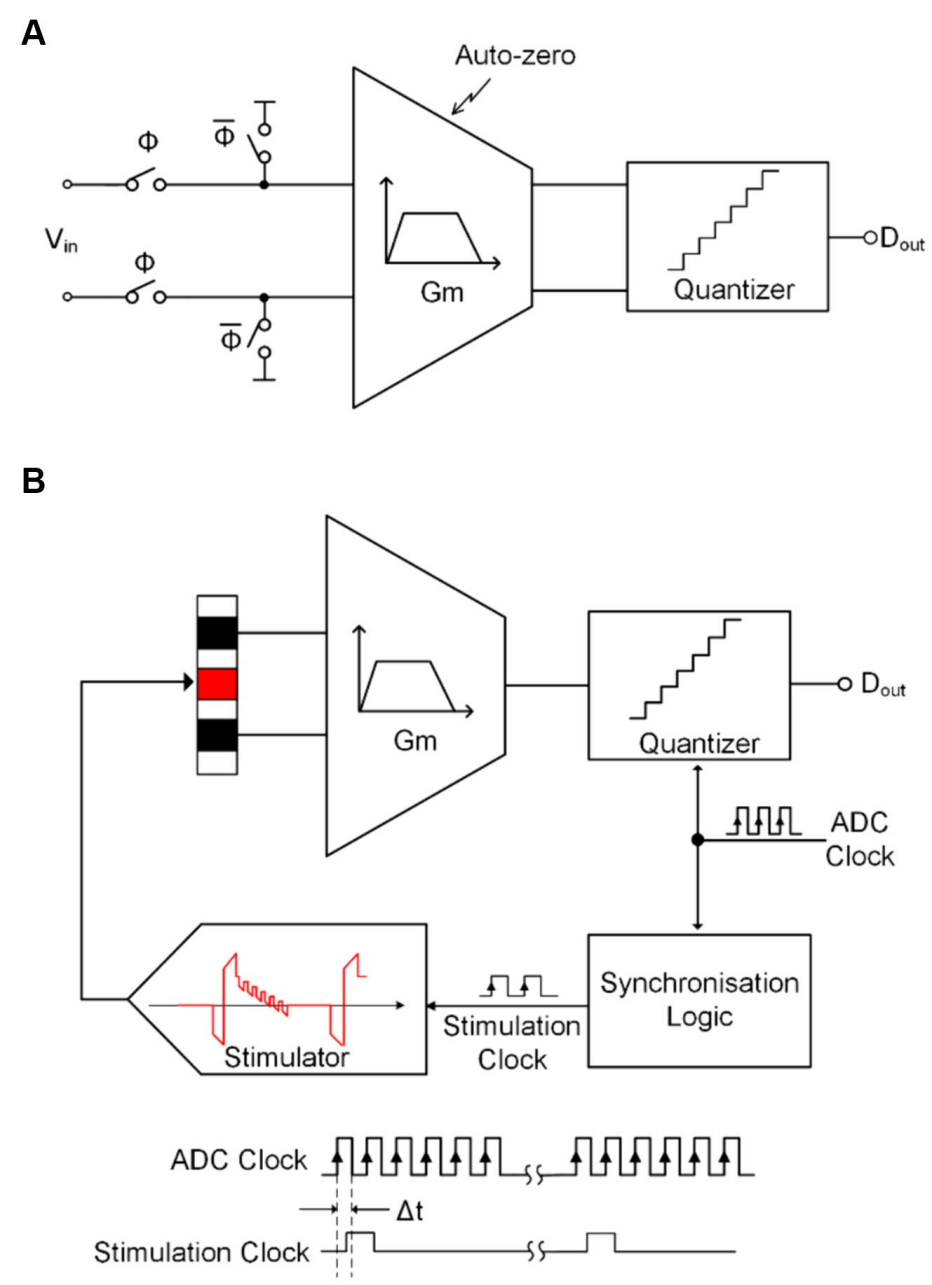
**(A)** The block diagram of the conventional blanking technique. **(B)** The block diagram of blanking with synchronization logic.

Figure 7A shows one of the structures that implements blanking at the input end of the neural amplifier (Bi et al., 2020; Huang et al., 2023; Qiu et al., 2023) achieve the shielding function of artifact signals by generating a synchronous clock during the stimulation cycle to control the sampling switch at the input end. It is worth noting that when controlling the on/off of the sampling switch, a certain time interval $t_{\mathrm{gap}}$ should be preserved. Therefore, the entire blanking time $t_{\mathrm{blanking}}$ can be expressed as:


$$t_{\mathrm{blanking}}=t_{\mathrm{gap}1}+t_{\mathrm{cat}}+t_{ipg}+t_{ano}+t_{\mathrm{gap}2}$$

Where $t_{ipg}$ is the inter-phase gap time interval between negative and positive pulses in biphasic constant current stimulation (CCS) and $t_{\mathrm{cat}}\left(t_{ano}\right)$ is the negative (positive) stimulation pulse width.

Different from the method of shielding artifact signals at the input end, (Debarros et al., 2020) proposed a technology that simultaneously controls the ADC sampling clock and stimulation clock through a synchronous logic unit to achieve the shielding of artifact signals. In this work, the stimulation pulses happen between two ADC samplings, so it guarantees the ADC holds the recoding to its previous voltage before each stimulation pulse, as shown in Figure 7B.

On the one hand, Blanking technology will cause the information of the stimulation cycle to be lost and it is difficult to recover quickly after the stimulation cycle; on the other hand, when the switch is on, kT/C noise will be introduced at the input end (Erez et al., 2010).

### 3.5 Soft-reset

Similar to Blanking technology, soft-reset technology also uses the periodicity of artifacts to shield them (Liu et al., 2016; Viswam et al., 2016; Shadmani et al., 2019; Erbsloh et al., 2021). The difference is that blanking technology performs shielding in the time domain, while soft-reset technology performs shielding in the frequency domain.

**Figure 8:**
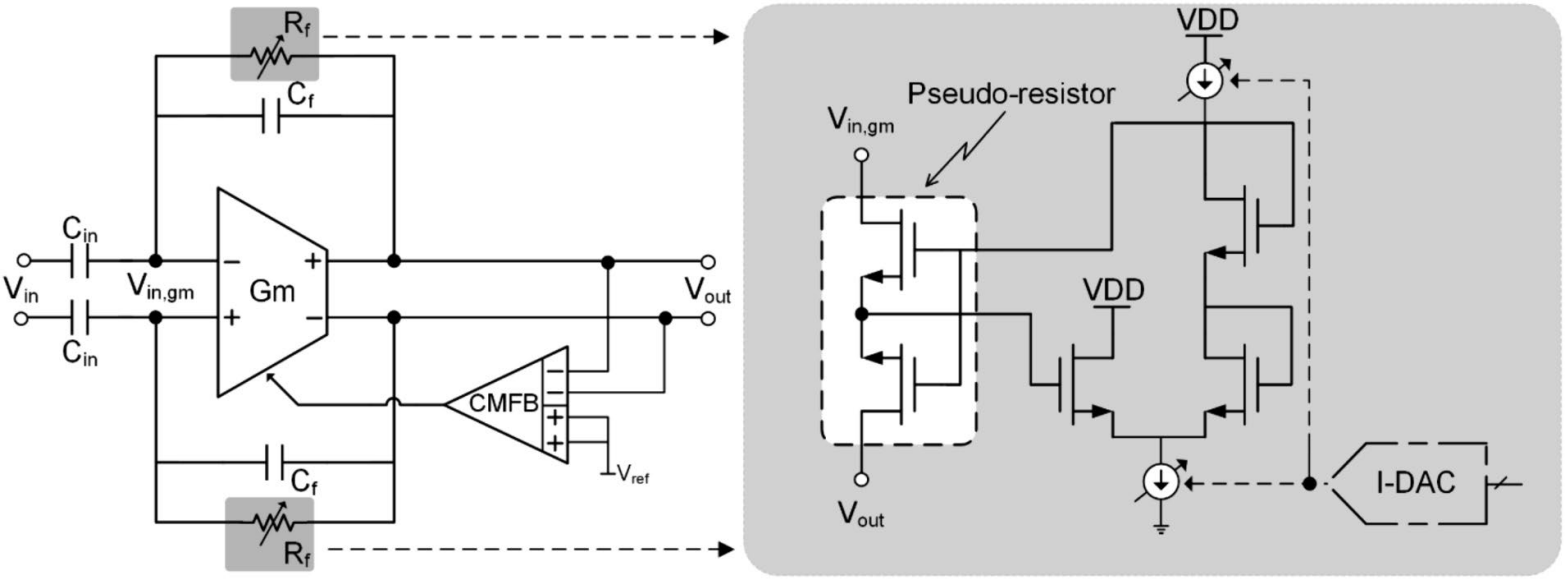
The block diagram of the soft-reset (pole-shifting) technique.

Figure 8 shows the schematic of soft-reset technology. It modifies the high-pass cutoff frequency by controlling the current in the pseudo-resistor in the capacitive feedback amplifier, thereby reducing the gain of the neural amplifier to avoid saturation. Therefore, this technology is also called pole-shifting. For a capacitive feedback amplifier, a pseudo resistor (Sharma et al., 2016; Guglielmi et al., 2020) is usually used to bias the DC operating point of the amplifier. At the same time, the pseudo-resistance will also introduce a low-frequency pole $p_{1}=1/C_{2}R_{f}$. During the stimulation cycle, the system will increase the adjustment current $I_{\mathrm{tune}}$ in the pseudo resistor to reduce the equivalent impedance, thereby increasing the high-pass cutoff frequency of the amplifier to the kHz level to reduce its gain. In addition, the time constant of the amplifier becomes very small at this time (approximately in the range of 100 μs (Erbsloh et al., 2021)). This allows the neural recording front end to quickly recover and function normally after the stimulation cycle ends.

Although soft reset (pole-shifting) technology has a faster recovery time than blanking technology, there is still a risk of losing important information. In addition, pseudo-resistor leakage current exists in practical applications, which will affect the DC operating point of the amplifier. Therefore, when using soft reset (pole-shifting) techniques, it is still necessary to consider the non-ideal factors introduced by pseudo-resistance during non-stimulation periods.

### 3.6 Signal-folding

When the system applies blanking technology or soft reset technology, the recorded neural signal waveform will lose part of the information during the stimulation cycle. The signal folding technology can fold the signals before and after the stimulation cycle to specific values through a specific reset circuit topology, and then restore the signal through a reconstruction algorithm (Chen et al., 2014).

**Figure 9:**
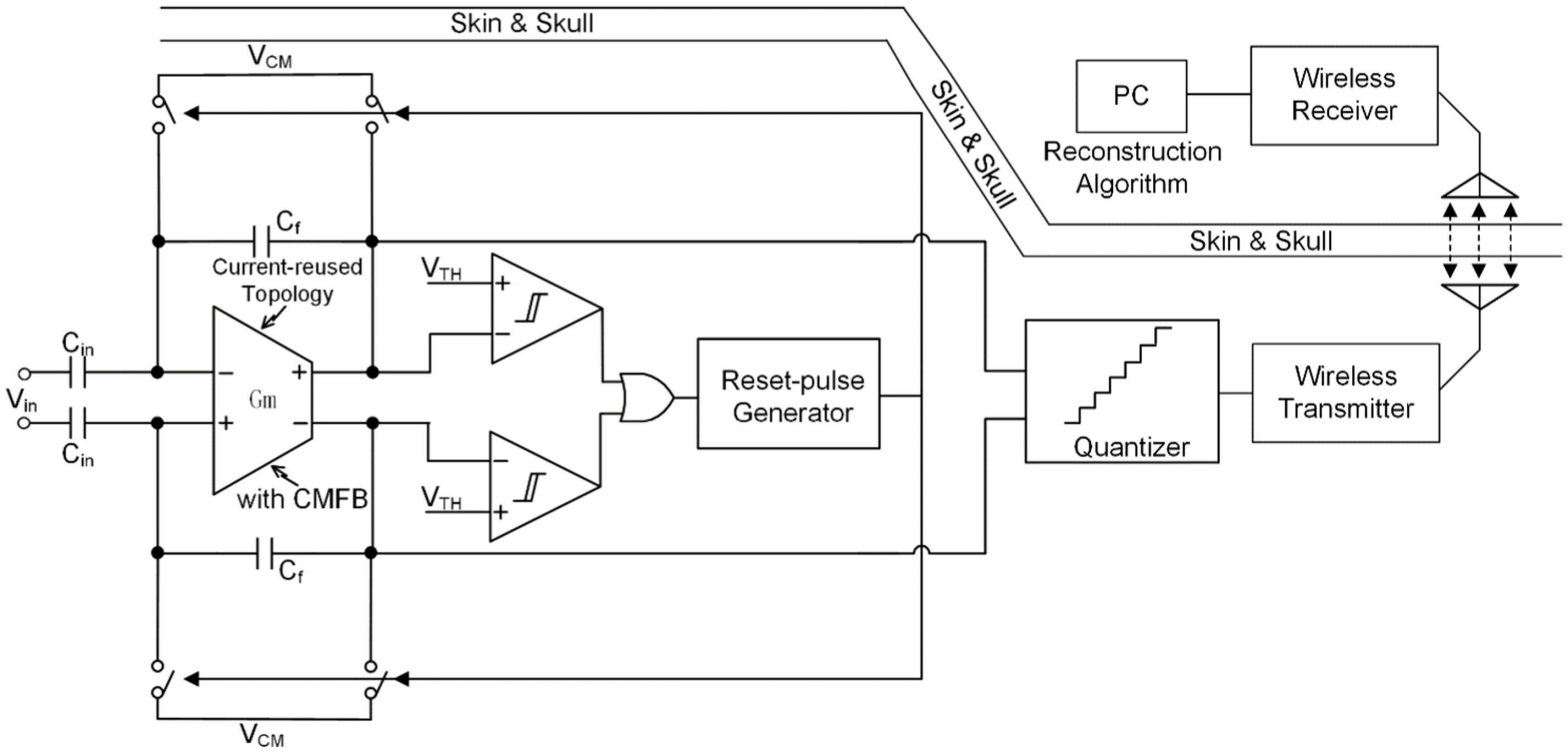
The block diagram of the signal-folding technique.

The signal folding concept is illustrated in Figure 9. Whenever at any time, the output signal falls below the threshold voltage $V_{th}=V_{CM}-△V_{th}$, the circuit will generate a narrow-reset pulse signal to set the input and output voltages of the amplify-er to the reference voltage. Before and after the reset cycle, the output signal is folded into a voltage range of $2△V_{th}$. In order to recover the amplified signal, a non-Nyquist reconstruction process is applied to the signal digitized by the ADC. This greatly relaxes the design requirements of the ADC.

Due to the slow settling time of the amplifier, signal-folding technology will still lose some information for a period of time after reset and cannot be recovered. In addition, the integration level of the system is greatly reduced by integrating a wireless communication module.

### 3.7 Adaptive filter

**Figure 10:**
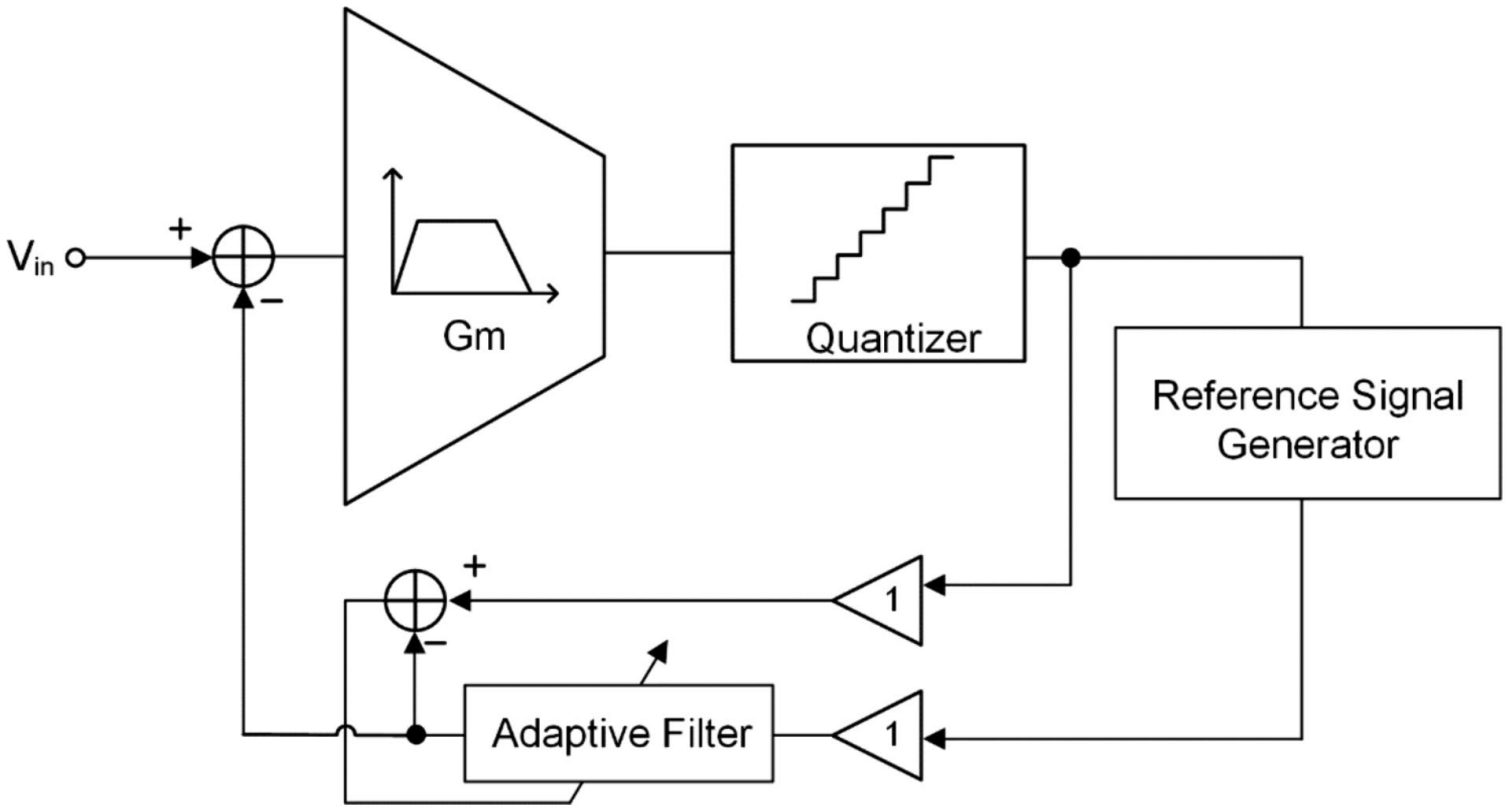
The block diagram of the fast stimulation artifacts rejection technique with adaptive filter.

Similar to adaptive active noise cancelation technique (Sugiyama et al., 2011; Deb et al., 2014), the fast simulation artifact rejection (FSAR) technique eliminates artifact signals at the NRFE input end in the form of negative feedback through adaptive filter and reference signal generator, as shown in Figure 10 (Samiei and Hashemi, 2021; Wang et al., 2021). If a timely approximation of the artifact signal (or replica artifact signal) can be obtained through the reference signal generator, the accurate artifact signal can be obtained through the adaptive filter and fed back to the input. In this way, the artifact interference signals contained in the input signal can be eliminated, leaving behind the desired neural signal.

Although NRFE can eliminate artifacts in digital domain by using adaptive filters, it can only eliminate DMA (or smaller amplitude artifacts) but will significantly reduce the DR of the system (Chandrakumar and Marković, 2017a,b,c).

### 3.8 CM cancelation path

**Figure 11:**
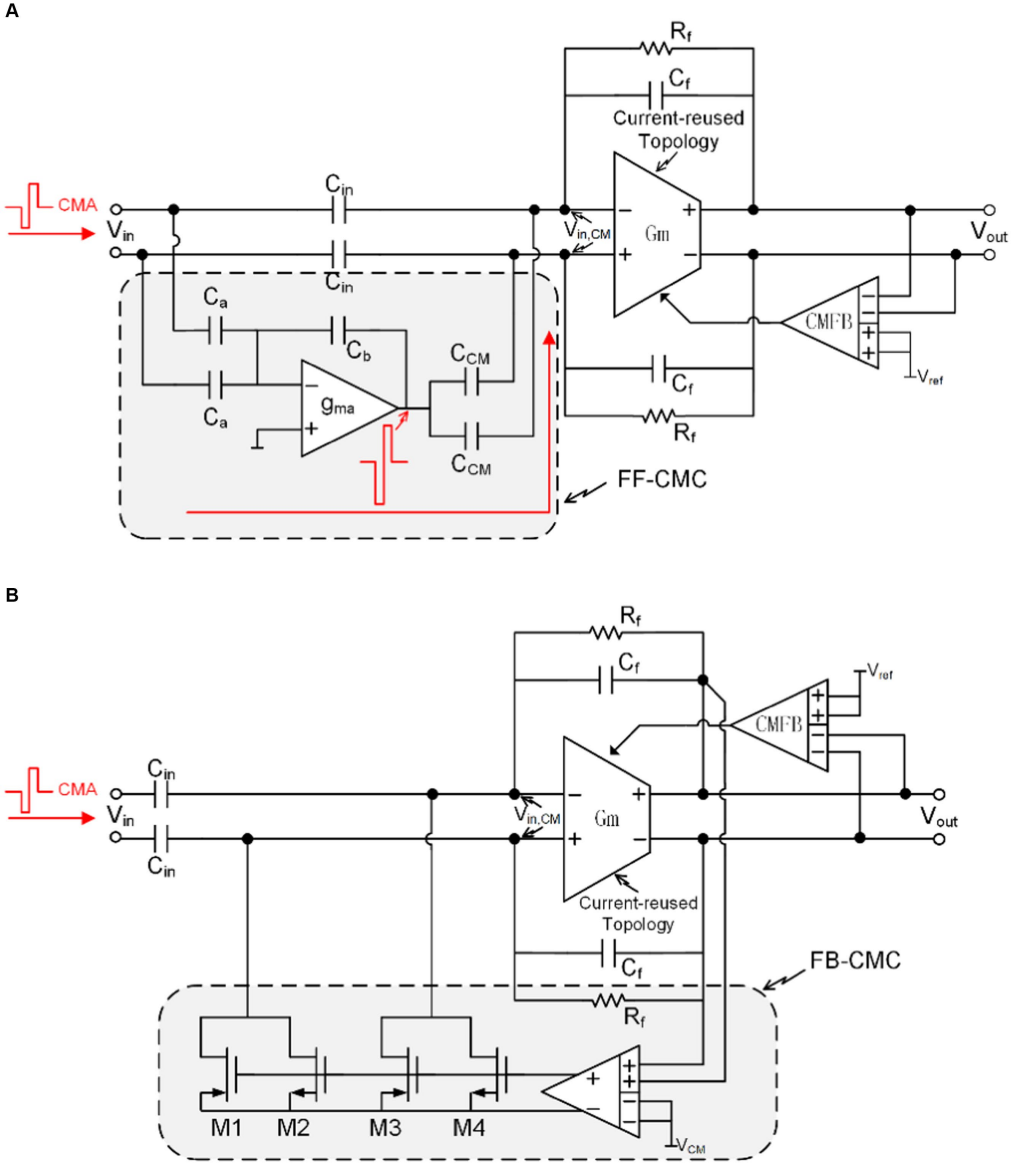
**(A)** The block diagram of the feedforward common-mode cancelation path. **(B)** The block diagram of the feedback common-mode cancelation path.

A CM feedforward topology can be used to eliminate the effects of CMA, referred to as feedforward CM cancelation (FF-CMC) (Chandrakumar and Marković, 2017a,b,c; Pham et al., 2022). The concept of FF-CMC path is shown in Figure 11A. The common-mode signals of the two recording electrodes in one channel are sensed and inversely amplified by a feedforward capacitive feedback adder ($g_{ma}$, $C_{a}$, $C_{b}$) and summed at the input of the amplifier $g_{m}$ through capacitor $C_{\mathrm{cm}}$. Among them, by rationally designing the sizes of the gain of the FF-CMC path ($A_{CM}$) and$C_{CM}$, the effects of CMA can be completely eliminated:


$$A_{CM}=2C_{a}/C_{b}$$

$$C_{CM}=C_{\mathrm{in}}/A_{CM}$$

To ensure accurate elimination of CMA, the bandwidth of the FF-CMC path needs to be much larger than k times the maximum frequency of the neural signal ($f_{\mathrm{signal},\max}$). This requirement can be expressed by the following formula:


$$g_{ma}\;.\;\frac{C_{b}}{2\pi \left(2C_{CM}+C_{b}\right)\left(2C_{a}+C_{b}\right)-C_{b}^{2}}>k.f_{\mathrm{signal},\max}$$

From this formula, we can get the design requirements of $g_{ma}$.

FF-CMC technology requires the capacitance in the path to be completely and accurately matched. Any $C_{\mathrm{in}}/C_{CM}$ mismatch will cause residual CMA to be introduced as DMA into the neural amplifier. This is the limitation of this technology. Fortunately, the neural amplifier $g_{m}$ is immune to smaller CM swing (<20 mV). Therefore, it is necessary to use common centroid technology in the layout to achieve a smaller mismatch ratio (<0.1%). In addition, the presence of the capacitors$C_{CM}$ leads to an increase in the input-referred noise of the front-end ($v_{n,\mathrm{input}}$), as shown by the following equation:

$$v_{n,\mathrm{input}}=v_{n}.\left(1+\frac{C_{\mathrm{in}}+C_{CM}}{C_{f}}\right)$$

$v_{n}$ is the input-referred noise of the neural amplifier $g_{m}$. Therefore, the capacitance needs to be appropriately selected so that its input-referred noise meets the system requirements.

Feedback-CMC (FF-CMC) can also adjust the input common mode signal in real time through negative feedback (Wang et al., 2021). As shown in Figure 11B, if the output common mode level is greater than the reference common mode level, M1 and M3 will be turned on to reduce the input common mode level, and if the output common mode level is smaller than the reference common mode level, M2 and M4 will be turned on to enhance the input common mode level.

### 3.9 Direct-conversion topology

All methods described previously require IA combined with ADC to quantify neural signals. With the development of neural signal processing algorithms in recent years, neural signals and artifacts can be distinguished directly from the signals recorded by the neural recording front-end. As a result, direct-conversion topologies were developed to replace the traditional neural recording front-end that combines IA with ADC (Lee et al., 2020). Direct quantification of artifact-containing neural signals requires neural recording front-ends with large DR, linearity, and signal-to-noise-and-distortion ratio (SNDR). Motivated by feedback-based approach published in (Muller et al., 2011; Kassiri et al., 2016), DSM quickly became the preferred structure for direct quantification applications (Jung et al., 2022a,b).

**Figure 12:**
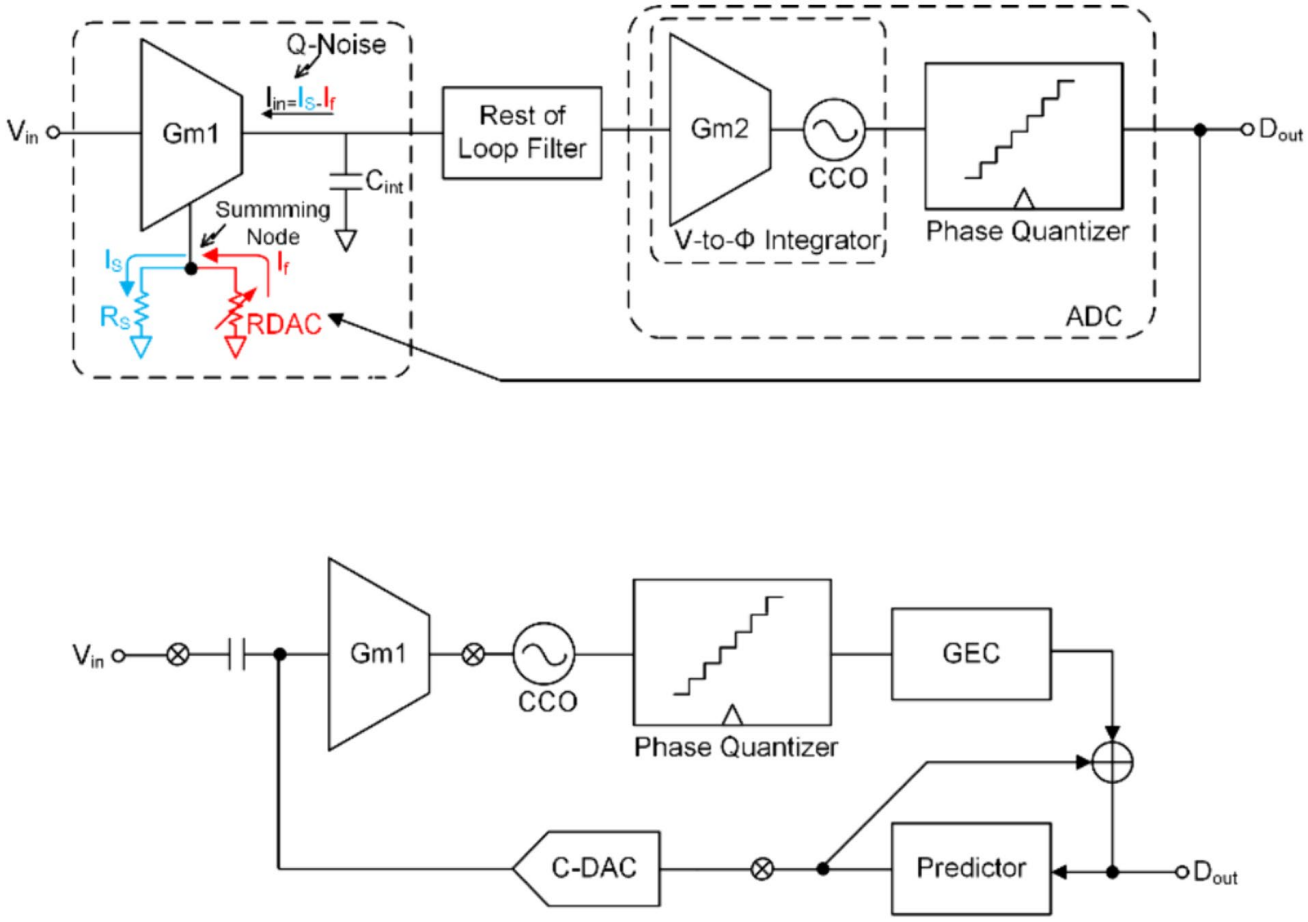
**(A)** The block diagram of the DC coupling CT-DMS ADC. **(B)** The block diagram of a DSM using a time-based quantizer and DPCM.

One of the topologies is derived from highly linear DC-coupled $G_{m}-C$ based CT-DSM for artifact-tolerant neural recording (Jeon et al., 2019; Lee et al., 2020). The conceptual block diagram of DC-coupled $G_{m}-C$ based CT-DSM with the resistive feedback digital-to-analog convertor (RDAC) is shown in Figure 12A. The DC-coupled DSM (Nikas et al., 2019; Moeinfard and Kassiri, 2022) has a higher input impedance than ac-couple DSM with chopper topology (Johnson et al., 2017; Bang et al., 2018; Zhao et al., 2019) and conventional $G_{m}-C$ based CT-DSM (Schoofs et al., 2007; Sarhangnejad et al., 2011; Huang et al., 2015) (implementing a summing node at the output of the integrator). The traditional way to improve the linearity of $G_{m}$ is to achieve it through source degeneration technology (Wattanapanitch et al., 2007), but this will still cause the output of Gm to saturate in the case of a larger input signal. This occurs because the current generated by the source degeneration resistor is much larger than the bias current (or input transistor current) and can only be improved by increasing the resistance of $R_{S}$. To address the limitation of source degeneration technology, real-time adjustment of its current variations can be achieved through the integration of a negative feedback loop. In order to improve the linearity of $G_{m}-C$, RDAC and $R_{S}$ are connected in parallel. In this way, CTDSM can be used to make the current $I_{S}$ generated by the input signal equal to the feedback current $I_{f}$ of the RDAC. Due to the feedback-assisted Gm linearization, the magnitude of the $I_{\mathrm{IN}}=I_{S}-I_{f}$ maintains within the LSB of feedback DAC current even with a large input voltage, which improves the linearity of the $G_{m}-C$ integrator significantly (Lee, C. et al., 2020). A VCO-based integrator and quantizer, implementing with a $G_{m}$ cell followed by two current-control oscillators (CCOs) is implemented to improve energy and area efficiency.

Another topology applies digital pulse code modulation (DPCM) as a predictor that exploits correlation between adjacent samples of the input signal (Huang and Mercier, 2020, 2021), as shown in Figure 12B. The prediction of DPCM significantly reduces the input signal swing to improve the linearity of $G_{m1}$ and CCO.

### 3.10 Artifact template subtraction algorithm

While the aforementioned technologies address artifacts at the hardware system, it should be noted that no hardware system can comprehensively resolve or formulate the theory of large artifact removal in same-electrode stimulation and recording. Additionally, complete hardware systems entail stringent system requirements and pose challenges in adapting to diverse application scenarios. Therefore, post-processing artifact suppression technology is needed to further process the neural signals in software system (or called algorithm) collected by the hardware system.

**Figure 13:**
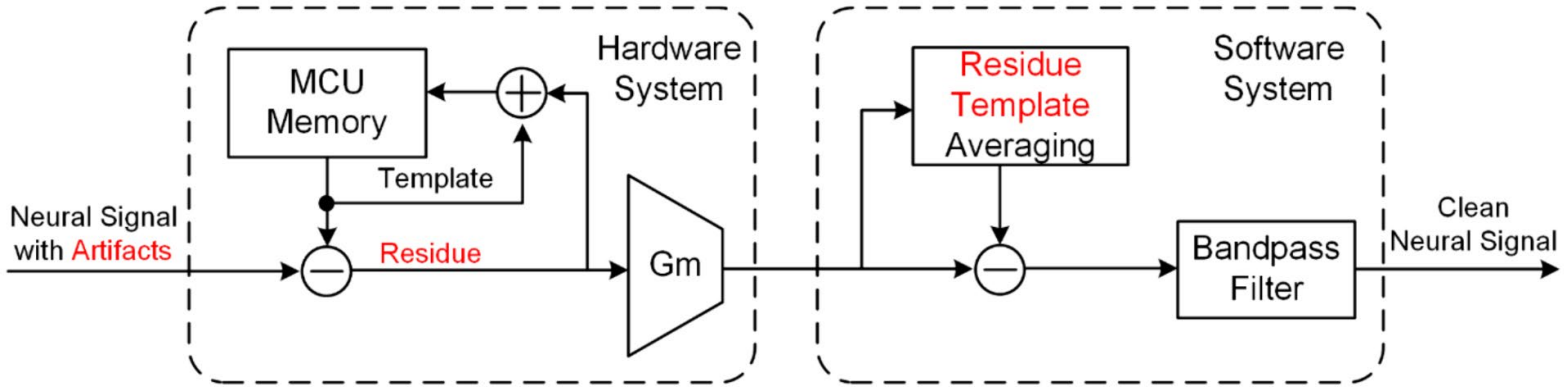
The block diagram of an online artifact cancelation in same-electrode system using combined hardware and software system.

Template subtraction (Culaclii et al., 2016, 2018; Pérez-Prieto et al., 2019) is a typical technology for eliminating artifacts in software systems, as shown in Figure 13. The neural signal finally output by the software system is obtained by subtracting a template representing the artifact waveform from the neural signal accompanying artifacts collected by the hardware system. The template can be generated by averaging the artifacts, polynomial function-fitting, and filtering based on wavelet methods and Hampel identifiers. The initial template is stored by the iterative hardware loop, then the template is gradually updated with each recorded artifact difference until the template converges in the hardware resolution.

It is important to recognize that if the neural recording front-end becomes saturated, software approach may fail to accurately process the desired neural signals. Hence, the integration of this technology with a hardware system boasting a larger dynamic range, as shown in Figure 13, is imperative to effectively eliminate artifacts, as demonstrated in (Culaclii et al., 2016, 2018).

## 4 Summary and classification

We have categorized all the technologies listed above, some technologies are only applied to MA, some technologies are only applied to SA, and some technologies are applied to both MA and SA, as shown in Table 2.

**Table 2:** Classification of the above anti-artifacts technologies.

Technology	MA	SA	MA and SA
Current compensation technology		√	
Moderate gain recording amplifier with high resolution ADC			√
Adaptive gain control			√
Blanking		√	
Soft-reset		√	
Signal-folding	√		
Adaptive filter	√		
CM cancelation path		√	
Direct-conversion topology		√	
Artifact template subtraction algorithm		√	

The previous section listed 10 technologies related to eliminating the impact of artifacts, and most of them have become the core technologies for addressing the closed-loop brain-computer interface artifact problem in recent years. We have selected 7 of the most representative articles in the past few years for comparison (as shown in Table 3). The technologies they apply encompass all the above-mentioned methods for mitigating the impact of artifacts.

**Table 3:** Comparison of the related anti-artifacts works.

			Chandrakumar and Marković (2018)	Jung et al. (2022a,b)	Qiu et al. (2023)	Liu et al. (2016)	Chen et al. (2014)	Wang et al. (2021)	Lee et al. (2020)
Process (nm)			40	180	180	180	180	180	110
Topology	MA		CT-DSM	AGC	N/A	N/A	Signal-folding	FSAR	DC coupling CT-DSM
	SA	CMA	FF-CMC	AGC	Blanking	Soft-reset	N/A	FB-CMC	DC coupling CT-DSM
		DMA	CT-DSM					FSAR	
SA suppression range (mV)		CMA	700	max: 1600	5000	N/A	3	1,500	300
		DMA	200					N/A	
Recovery time (μs)			N/A	N/A	<150	N/A	N/A	500	N/A
Supply (V)			1.2	1.2 (A)	1.8/5/10	0.9	1	1.5	1
				0.8 (D)					
Power per channel (μW)			7.3	9.8 (A)	N/A	56	4.53	1.48	6.5
				13.6 (D)					
Gain (dB)			17.9	1,8–256 (7 steps)	34.4/59.5/68.9/79.4	40	54.2	14–44	N/A
BW (Hz)			1–200 (BW1)	1-5 k	0.1–8 k	0.3–7 k	1–5.7 k	0.1–1 k	10 k
			1–5 k (BW2)						
Input range ($V_{\mathrm{pp}}$)			1.77	1.6	N/A	N/A	N/A	N/A	300 m
IRN ($\mu V_{rms}$)			2.9	min: 6.1	4.09	4.57	min: 1.22	2.16	95 nV/$\sqrt{\mathrm{Hz}}$^*^
NEF			N/A	min: 9.5	6.88	4.77	min: 3.03	2.62	9.3
Input impedance (GΩ)			1.52	N/A	1.06–0.0106 (10–1 kHz)	N/A	N/A	>2.2	>0.0133
SNDR (dB)			86 (BW1)	max: 70.1	N/A	N/A	max: 43	N/A	80.4
			78 (BW2)						
ENOB (b)			15.2	max: 11.4	11	max: 9.1	8	N/A	15
DR (dB)			90 (BW1)	99.5	106.8	N/A	66	N/A	81
			81 (BW2)						
FOM (dB)^*^			160.4 (BW1)	185.2	N/A	max: 34.2	N/A	N/A	172.3
			166.4 (BW2)						

*$FOM=\mathrm{SNDR}+10\log\left(BW/\mathrm{Power}\right)$.

## Author contributions

WC: Conceptualization, Data curation, Formal analysis, Funding acquisition, Investigation, Methodology, Project administration, Resources, Software, Supervision, Validation, Visualization, Writing – original draft, Writing – review & editing. XL: Data curation, Formal analysis, Funding acquisition, Validation, Writing – review & editing. PW: Data curation, Formal analysis, Methodology, Writing – review & editing. ZC: Funding acquisition, Investigation, Project administration, Writing – review & editing. YC: Conceptualization, Resources, Supervision, Writing – review & editing.
